# Design Rules for Hybrid Additive Manufacturing Combining Selective Laser Melting and Micromilling

**DOI:** 10.3390/ma14195753

**Published:** 2021-10-02

**Authors:** David Sommer, Babette Götzendorfer, Cemal Esen, Ralf Hellmann

**Affiliations:** 1 Applied Laser and Photonics Group, University of Applied Sciences Aschaffenburg, 63743 Aschaffenburg, Germany; david.sommer@th-ab.de (D.S.); ralf.hellmann@th-ab.de (R.H.); 2 Applied Laser Technologies, Ruhr-University Bochum, 44801 Bochum, Germany; esen@lat.rub.de

**Keywords:** hybrid additive manufacturing, high-speed milling, selective laser melting, construction rules

## Abstract

We report on a comprehensive study to evaluate fundamental properties of a hybrid manufacturing approach, combining selective laser melting and high speed milling, and to characterize typical geometrical features and conclude on a catalogue of design rules. As for any additive manufacturing approach, the understanding of the machine properties and the process behaviour as well as such a selection guide is of upmost importance to foster the implementation of new machining concepts and support design engineers. Geometrical accuracy between digitally designed and physically realized parts made of maraging steel and dimensional limits are analyzed by stripe line projection. In particular, we identify design rules for numerous basic geometric elements like walls, cylinders, angles, inclinations, overhangs, notches, inner and outer radii of spheres, chamfers in build direction, and holes of different shape, respectively, as being manufactured by the hybrid approach and compare them to sole selective laser melting. While the cutting tool defines the manufacturability of, e.g., edges and corners, the milling itself improves the surface roughness to Ra < 2μm. Thus, the given advantages of this hybrid process, e.g., space-resolved and custom-designed roughness and the superior geometrical accuracy are evaluated. Finally, we exemplify the potential of this particular promising hybrid approach by demonstrating an injection mold with a conformal cooling for a charge socket for an electro mobile.

## 1. Introduction

Additive manufacturing (AM), especially selective laser melting (SLM), is continuing to move towards broader acceptance in industry in manifold applications. However, at the same time fundamental limitations remain a challenge, one of them being the surface roughness of SLM built parts, which may be of unsatisfactory quality, in turn restricting usage of such parts in applications that demand close-fitting [1,2], exigent fatigue strength [3], or distinct sterilizability for bio-medical appliances [4]. Thus, SLM parts are typically post-processed by, e.g., either shot-blasting, milling, turning or chemical treatment in order to meet the desired requirements [5,6]. This necessity has led to the development of different hybrid processes and machine concepts (not only for SLM), combining additive techniques with conventional machining. For example, in several approaches CNC-machines are combined with laser wire deposition or metal arc welding to form a hybrid process [7,8,9,10,11]. Another hybrid approach that can vanquish the afore mentioned challenges, combines SLM with in-situ milling [12], allowing for the freedom of design offered by additive manufacturing [13,14,15] combined with the geometric accuracy and surface quality of milling [16,17,18] within a single, automated process.

The advancement of hybrid machines that combine additive and subtractive processes indeed paves the way towards new concepts in both product design and manufacturing [8], thus enabling the construction of innovative components that had previously been beyond reach [5,9]. However, this also demands for a throughout understanding of the fundamental properties of these new technologies, their restrictions and requirements.

While most of the research on hybrid additive manufacturing focusses on the development of processes and parameter optimization [19,20], on materials and product design [9,21,22], studies on appropriate design guidelines are, however, rare. Nonetheless, for the advancement of any AM technology, from a design engineering point of view, specific design rules and an elaborate selection guide for an appropriate additive manufacturing are a prerequisite for successful industrial implementation. Such rules have recently been specified for, e.g., fused deposition modelling [23,24], selective laser sintering [24], SLM [25,26,27,28], electron beam melting [29] and binder jetting [30]. For a hybrid AM approach as addressed here, however, such design rules are not yet available.

This particular hybrid SLM process itself has previously been demonstrated in different application oriented case studies, such as, e.g., the manufacturing of dental implants. In Ref. [31,32], the generation of an Akers clasp with improved fitting accuracy, retentive force and surface roughness of the cobalt-chromium alloy clasp as compared to a cast clasp is reported. Ohkubo et al., in turn, manufacture titanium removable partial dentures [33].

However, in these studies details about the fabrication process, fundamental properties as well as general design limitations are not discussed. Without this fundamental knowledge, the described hybrid process may not be used to full capacity. Wüst et al. report on an experimental study to optimize the surface roughness of hybrid additive manufactured parts [34], with particular focus on optimization of both SLM and milling to improve the vertical and horizontal surfaces. As the described hybrid process bears the opportunity to adapt the surface properties in dependence of the local required quality, it is crucial to define precise threshold values.

Against this fundamental background, we report on a comprehensive study to define the geometrical accuracy and design limits of selected fundamental geometrical elements to form a catalogue of design rules and compare these to the sole SLM process. In particular, we design 3D models of walls, cylinders, different inner angels, inclinations, overhangs, notches, inner and outer radii of spheres, chamfers in build direction, and holes of different shape and compare these structures in term of geometrical accuracy and feasible minimum dimensions to sole SLM. This shall ultimately support design engineers to purposefully apply hybrid additive manufacturing. Further the role of oversized grain by powder characterization of the performed maraging steel (1.2709), a low-carbon and high-nickel alloy, is analysed. Such aspects that have not been comprehensively reported before, yet define the fundamental basis for any further process optimization and component development.

## 2. Materials and Methods

### 2.1. Machine and Process

**Figure 1:**
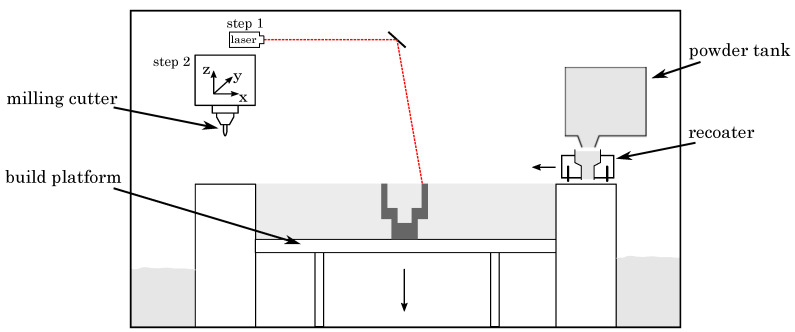
Hybrid manufacturing process.

For the study of the hybrid manufacturing approach, we employed a Lumex Avance-25 (Matsuura Machinery GmbH, Wiesbaden, Germany), combining SLM and three-axis high speed milling, as schematically illustrated in Figure 1. The SLM process (step 1) follows the conventional procedure of additive manufacturing, embracing the layer-wise building of components by the selective laser additive process, which has been extensively examined by various studies [35,36,37]. The high speed milling (step 2) is performed with a 3-axis milling system, machining the contour surfaces between a preset number of selectively laser molten layers (typically ten).

For SLM, the machine is equipped with a $P_{L}=500$ W yttrium fiber laser YLM-500 (IPG Laser GmbH, Burbach, Germany) with an operating wavelength of λ=1070 nm and a spot size of $d_{\mathrm{spot}}=200\;\mu$m at focus position. The machine processes under nitrogen atmosphere with less than 3.0% oxygen level. To maintain the machined part at an elevated temperature, as to avoid deformation by curling due to residual stress, the build platform is kept at $\vartheta_{\mathrm{plate}}=50$ ${}^{\circ }$C. The maximum build volume is 250 mm × 250 mm × 185 mm (width × depth × height). The study is performed processing maraging tool steel 1.2709. As the focus of this study is on design rules, we applied previously optimized, standard process parameters for the SLM (see Table 1) [34].

The three-axis milling system is basically not distinct from standard industrial high speed milling machines. The high speed spindle operates with a maximum of 45,000 revolutions per minute, working with a maximum turning moment of 1.31 Nm, accessing a tool magazine with 20 different tools. However, it is worthwhile to note, that compared to a conventional milling system no cooling lubricant can be used while milling within the powder bed. Thus, the high speed milling process constitutes a dry machining concept, facing the challenges like, e.g., exceeding temperature conditions or increased tool wear characteristics [38]. In our study standard process parameters are used (see Table 2). Please note, the used milling cutters are solid carbide cutting tools with a nano alloy composed of aluminium, titan and silicon for the reduction of wear characteristics (Mitsubishi Materials Corporation GmbH, Meerbusch, Germany).

**Figure 2:**
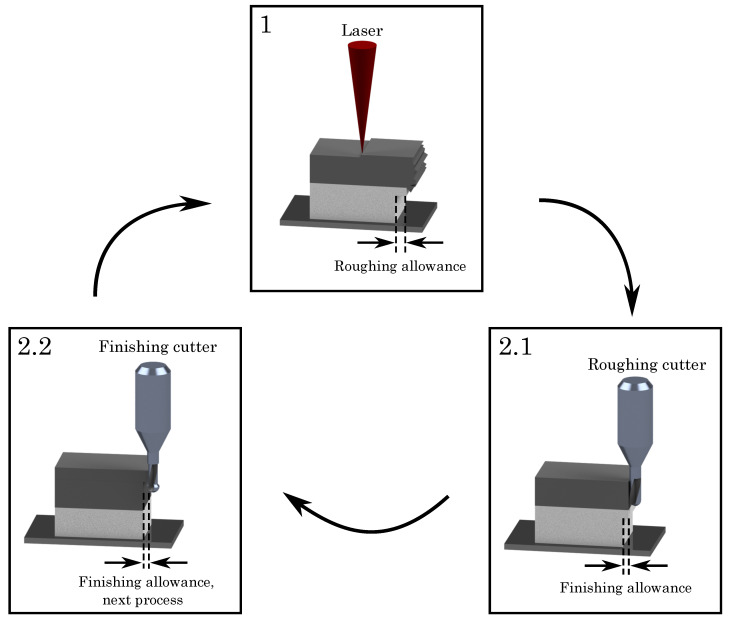
Two-stage milling process of hybrid manufacturing.

The milling process is, as mentioned before, directly integrated into the additive process, implying that the SLM process is disrupted after several layers for the milling of the previous built surfaces. Figure 2 depicts the single steps of the hybrid additive manufacturing approach. The SLM builts, in the standard process, ten layers with a thickness of $h_{\mathrm{layer}}=50\;\mu$m each, introducing a material allowance for the milling process (cf. Figure 2). The milling process is partitioned into a roughing and a finishing step using two different milling cutters. The two steps of the milling process gradually remove the material allowance of $d_{x/y}=150\;\mu$m which is added on the nominal constructed geometry by the SLM. The cutting depth of the roughing cutter represents 120 μm, getting removed working downwards the geometry, starting at the last built layer. Afterwards, the finishing cutter detaches the remaining 30 μm of the allowance (cf. Table 2), providing the final dimensions and a smooth surface, thus warranting the final quality of the build part.

However, it is worthwhile to note that the finishing cutter starts millling from the bottom to the top and also terminates beneath the last built layers, sparing several layers for the next process cycle (cf. Figure 2 (2.2), where the finishing cutter leaves five layers). Due to the selective laser melting, a thermal gradient within the built part developes, as the top layers retain a higher temperature compared to lower built levels, where the heat has dissipated via the subjacent parts or support structures. As it has been shown by [25,39,40], under such thermal conditions it is preferential to start milling from the bottom to the top and to also stop finishing beneath the upper surface, leaving several layers beyond. By virtue of this geometrically shift, the milling process avoids thermal deformations and geometrical deviations, warranting a high surface quality.

### 2.2. Powder Characterization

In this study, we processed 1.2709 maraging steel II (Matsuura Machinery GmbH, Wiesbaden, Germany), which is a low-carbon and high-nickel steel. The term maraging refers to the fact that the material has a martensitic microstructure and that it can be hardened and its strength be increased by aging. Due to its high strength, the alloy is used in tooling, structural, and aerospace applications [41]. The chemical composition of this powder is as listed in Table 3.

The particle size distribution of new and recycled powder was analyzed by a Camsizer X2 based on dynamic digital image analysis methods according to ISO 13322-2 (Retsch Technology GmbH, Haan, Germany).

### 2.3. Optical Characterization Tools

Different optical characterization tools are used to measure the shape deviation of the test geometries and for closer inspection of small parts. In particular, the geometrical deviation between the digitally designed and physically realized specimens, i.e., verification of the shape accuracy, was determined by stripe line projection using AtosCore 3D-scanner and employing shape variance analysis using ATOS Professional V8-SR1 software (GOM GmbH, Braunschweig, Germany).

For closer inspection of small parts and for validaton of the measured shape deviations, the digital microscope DVM6 (Leica Microsystems GmbH, Wetzlar, Germany) with a PlanAPO FOV 12.55 (maximum zoom of 675:1) is used. The specimens are captured with several images, using the z-stack function to generate a good depth of field.

## 3. Results and Discussion

### 3.1. Powder Characterization

**Figure 3:**
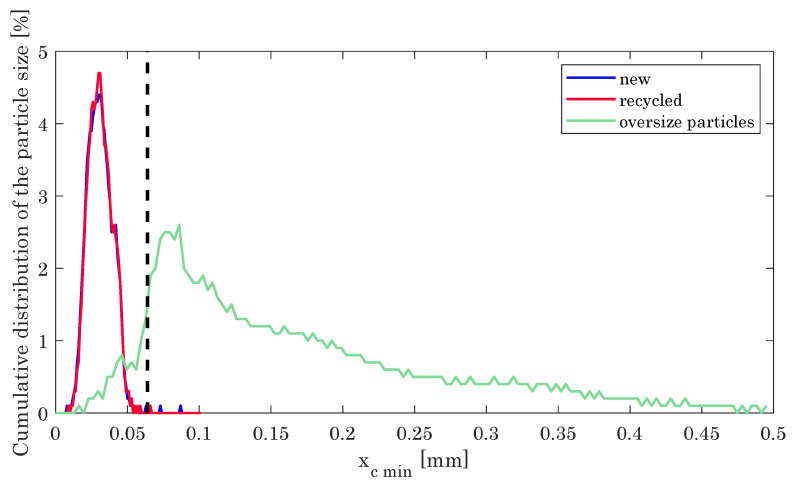
Powder distribution of new powder, recycled powder and the oversize particles.

During the SLM process, the particle size distribution of the employed powder and the shape of the individual particles typically change and get deteriorated due to adherence and mechanical deformation of particles and grains. This in turn necessitates sieving of used powder to ensure proper size distribution with good flowability. For the hybrid process discussed here, however, chips of milled material and abrasive wear of the milling cutters result in additional debris, in turn influencing the powder bed. Figure 3 compares the particle size distribution of new and recycled, i.e., sieved powder, as well as the oversize particles originating from adhered powder, milled material and wear debris after sieving. For sieving we used a mesh size of $d_{x}=63$ μm (cf. vertical line in Figure 3).

Apparently, the pristine and the recycled powder exhibit negligible differences with almost identical size distribution peaking at 31 mm. The sieved oversized particles, however, reveal a broader size distribution extending beyond 500 μm.

**Figure 4:**
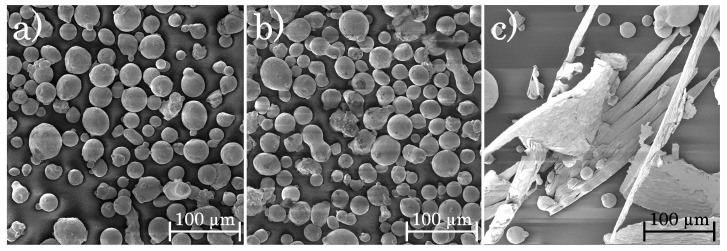
SEM images of (**a**) new, (**b**) recycled and (**c**) oversize particles.

For comparison, Scanning Electron Microscope (SEM) images of the pristine, recycled and of the oversized particles are shown in Figure 4, confirming that new and recycled powder exhibit similar shape. The oversize particles, however, clearly reveal larger chips of different size and shape which can be associated to splints of the cutter and flakes of the milled steel.

### 3.2. Geometric Aspects

While the overall potential of AM, in general, as well as design, material and process limitation have been subject of numerous studies, from an engineering point of view, specific design rules are a necessity and prerequisite to fully exploit the opportunities of each additive and hybrid manufacturing technology. Such design rules may outline aspects of geometrical accuracy and accessible surface roughness. For the hybrid approach under study, however, such design rules are yet unpublished.

**Figure 5:**
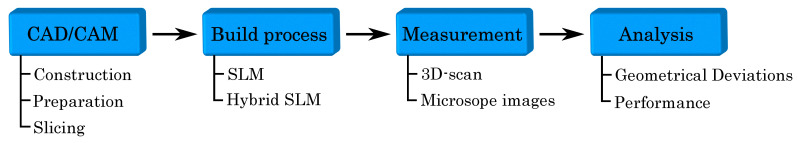
Methodical approach of the systematic creation of construction rules.

**Figure A1:**
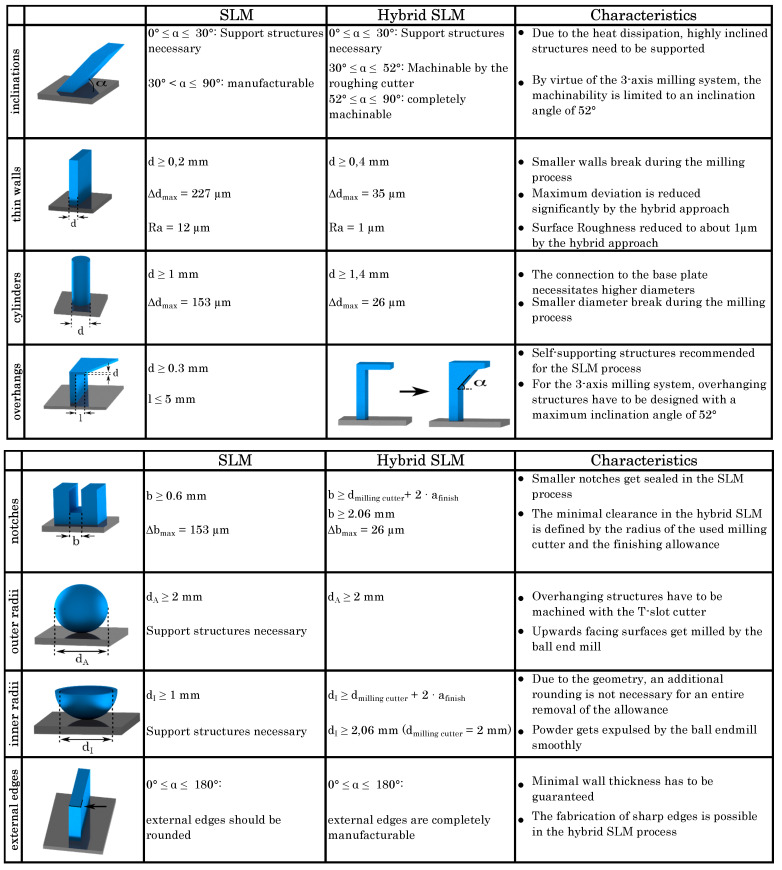

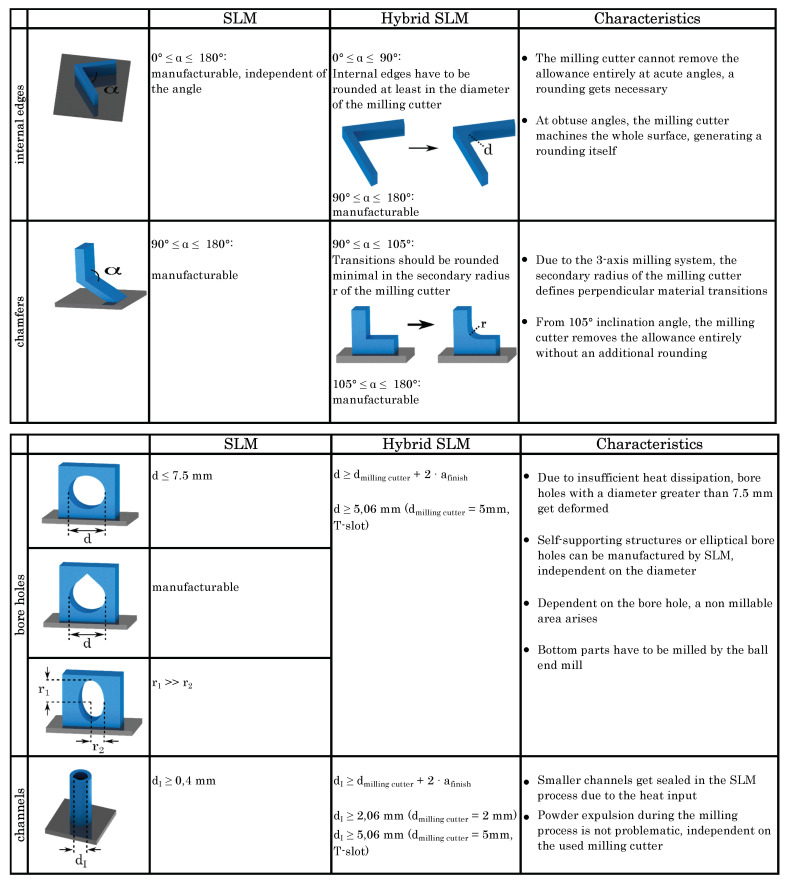
Design Guideline for the sole SLM and the hybrid additive Manufacturing.

In general, the methodical approach for the systematic creation of construction rules is realized similarly for all geometrical structures, as schematically illustrated in Figure 5. For every geometry, the construction has to be prepared in a CAM-software and sliced for the building process. After that, for both, the sole SLM and the hybrid process, a batch of specimens is manufactured, typically consisting 35 to 50 specimens. The geometrical accuracy is measured with the 3D-scanner and depicted in a false colour representation, additional measurements are carried out with a microscope and images are taken. Concluding, the analysis of the geometrical deviations and the performance of the milling system is executed and the findings are represented in a construction rule for both parts of the process and summarized in Figure A1 at the end of the publication.

As one of the most challenging basic geometries, inclined structures, generally, pose limits to SLM processes. In order to exemplify the methodical approach of this study (cf. Figure 5), in the following it is thus comprehensively outlined and exemplified for the fabrication of inclined structures. Specifically for this geometry, the most peculiarities occur and the most intense analysis has to be realized.

#### 3.2.1. Inclined Structures

The manufacturing of inclined structures is one of the limiting criterions in the SLM process. To allow sufficient heat dissipation, inclinations have to be built with additional support structures to avoid thermal deformation [14,42,43,44]. Depending on material and process parameters, the minimum inclination angle for the manufacturing of unsupported structures is defined for SLM in several studies between 20${}^{\circ }$ [25,45,46] and 45${}^{\circ }$ [15,24].

**Figure 6:**
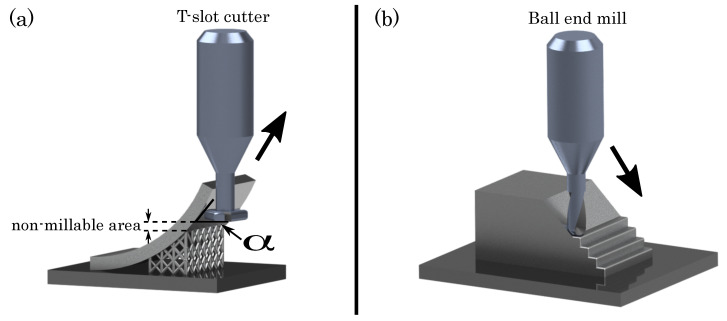
(**a**) Limitation of the hybrid manufacturing process of inclined structures, moving upwards (**b**) Removing of the staircase-effect by the milling cutter, moving downwards.

As a consequence, the primary limitation for the manufacturing of inclined structures in the hybrid process is given by the necessity of support structures in the SLM process. Another limitation is given by the three-axis milling system, enabling the milling of inclined structures with the use of T-slot milling cutters. The milling system permits a post-processing from an inclination angle of $\alpha =52^{\circ }$ upwards, determined by the geometry of the T-slot cutter (cf. Figure 6a). With that a non-millable area between the support structure and the finishing area arises.

Apart from the necessity of support structures, the geometrical accuracy of inclined structures is affected by the formation of the stair-case effect [47,48]. Due to the layerwise building process, the layers are geometrically shifted at the manufacturing of small inclination angles, as shown in Figure 6b. With the hybrid manufacturing approach, the staircase effect can be minimised, respectively excluded, and the surface is smoothened by the milling cutter after the building process [49].

**Figure 7:**
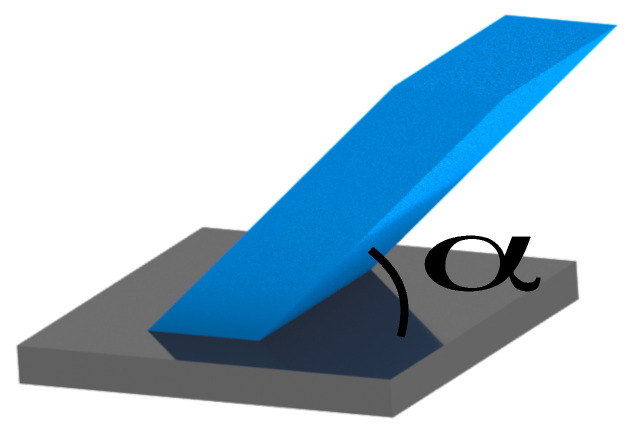
Test specimen for the study of inclined structures without support structures.

For the study of the geometrical accuracy and the machinability of inclined structures, walls with a varying inclination angle are manufactured, as depicted in Figure 7. The angles are specified in relation to the base plate and vary between 10${}^{\circ }$ and 90${}^{\circ }$ without the usage of support to examine the machinability of the bottom side of the specimens. The dimensions of the specimens are 10 mm in height, 6 mm in depth and 3 mm in width, remaining constantly.

**Figure 8:**
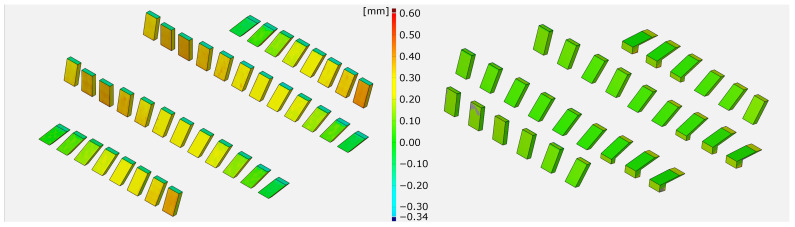
Comparison of SLM –built (**left**) and hybrid manufactured (**right**) inclinations (false colour representation).

Figure 8 illustrates and compares the SLM and hybrid built parts by a false colour representation provided by stripe line projection, indicating the dimensional deviation of the printed and constructed geometries.

Apparently, the geometrical deviation of inclined structures is, in general, larger for the SLM-built parts (colour code yellow and orange) as compared to the hybrid manufactured parts (colour code green). Please note, the SLM-built parts get manufactured without the additional material allowance for the milling process, the geometric forming is exclusively defined by the SLM process. The results reveal that the geometrical deviations of the SLM-built parts vary for different inclination angles. The top side of specimens show better accuracy in z-direction with nearly vertical shaped inclinations (10${}^{\circ }$–30${}^{\circ }$) than in x/y-direction with more horizontal aligned specimens (40${}^{\circ }$–90${}^{\circ }$). The hybrid manufactured parts show, as mentioned before, less geometrical deviations, persisting constantly, independent of the inclination angle. The colour code in the false colour representation does not vary significantly within the different specimens. In summary, the geometrical deviation of the SLM process varies between the z- and the x/y-direction, while the hybrid manufacturing approach shows omnidirectional consistent geometrical accuracy.

**Figure 9:**
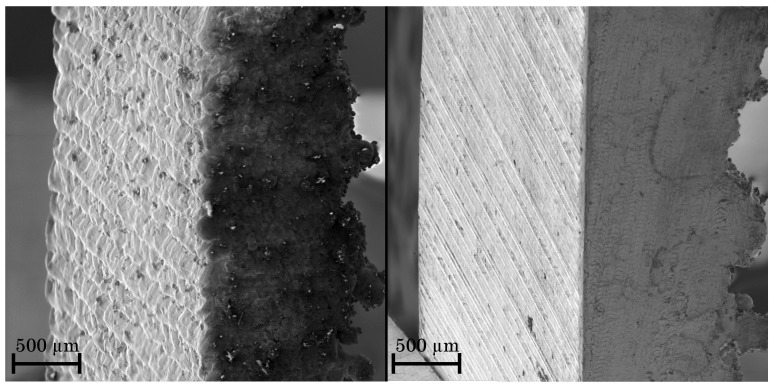
Comparison of highly inclined (inclination angle = 10${}^{\circ }$) SLM-built (**left**) and hybrid manufactured (**right**) parts.

In addition to the measurement of the geometric deviation by the stripe line projection, Scanning Electron Microscope (SEM) images of the surfaces are shown in Figure 9 to characterize the staircase-effect of highly inclined parts. The figure depicts the top and the front side of specimens with 10${}^{\circ }$ inclination angle; the unmachined bottom side is presented shortly on the right side of the specimens. The SLM built part on the left side of the figure exhibits misalignments between the single layers caused by the additive process, while the hybrid manufactured part on the right side shows a smooth surface, the staircase-effect itself is not identifiable and is excluded by milling.

**Figure 10:**
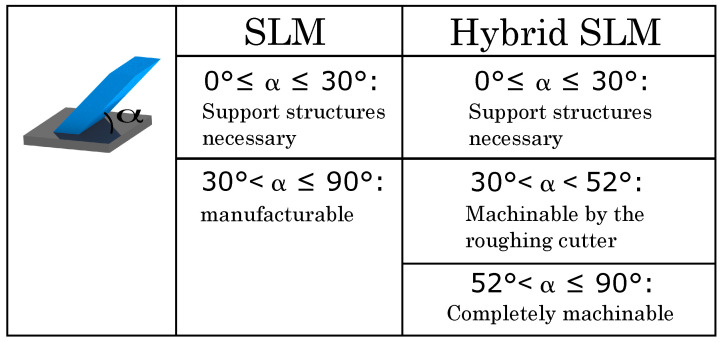
Contruction rule for the manufacturing of inclined structures.

From a design engineering point of view, this concludes a design rule for the hybrid SLM process, which is shown in Figure 10. For both, the sole SLM and the hybrid SLM process regions of inclination angles are specified considering the necessity of support structures and the machinability by the milling cutters.

#### 3.2.2. Wall Thickness

A fundamental geometrical element of any construction that has to be reliably fabricated by SLM is a rectangular shaped wall, for which SLM typically has its limits in terms of wall thickness. The lower limit is defined by the laser spot size [50,51] plus potentially wall broadening due to lateral adherent powder [52].

In the SLM process the minimum printed wall thickness is d=200μm, which appears to be governed by the laser spot diameter ($d_{\mathrm{spot}}=200\;\mu$m in our case) rather than the particle size [48]. It is also lower as previous reported construction rule studies for pure SLM, reporting minimum feasible geometrical dimensions between d=400μm [15,25] and d=600μm [24].

**Figure 11:**
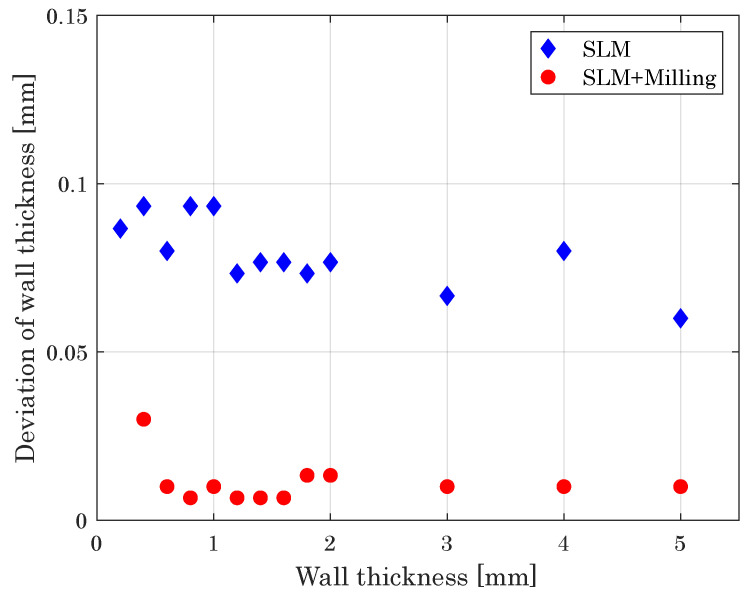
Deviation of wall thickness versus intended wall thickness, giving a measure of the dimensional accuracy of the processes.

The smallest wall thickness achievable with the hybrid system, in turn, is 400 μm. Thinner SLM built walls break under the milling process conditioned by the impact of the milling cutter. The milling cutter induces vibrations of the thin walls and by turning around the corner, the walls with smaller thickness get destroyed. Figure 11 compares the measured thickness deviation as a function of wall thickness for both the SLM and hybrid built parts. While the thickness deviation for SLM built parts is between 0.06 mm and 0.095 mm, the additional milling process improves the thickness deviation significantly. With a deviation of about 10 μm for thickness d≥600μm, the hybrid milling approach can eliminate the geometrical inaccuracy of the SLM process, one of its biggest disadvantages compared to conventional manufacturing methods [5].

#### 3.2.3. Cylinder Diameter

**Figure 12:**
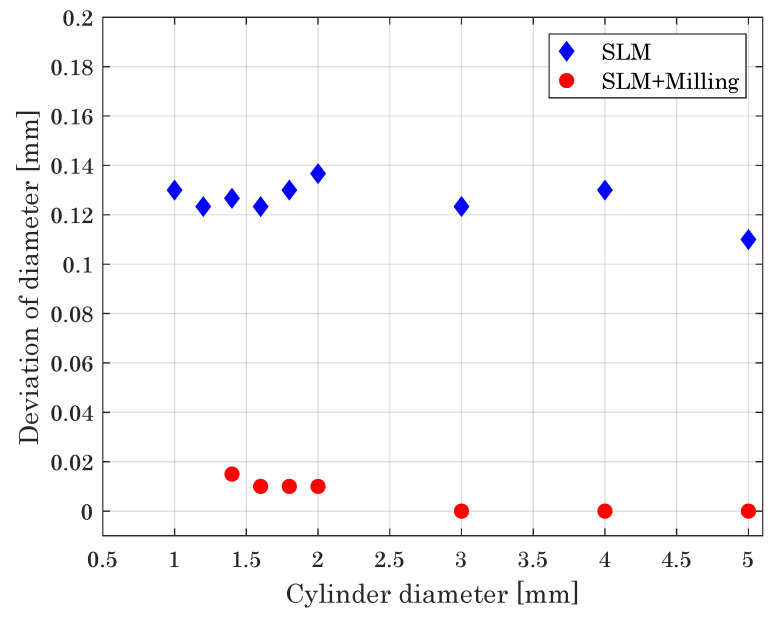
Deviation of cylinder diameter versus intended cylinder diameter, giving a measure of the dimensional accuracy of the processes.

Beyond thin walls, as a further fundamental geometrical element, we evaluated cylinders with respect to the minimum diameter achievable by the hybrid manufacturing approach (Figure 12). The minimum diameter is expected to be higher than the minimal printable wall thickness. Due to the smaller base area, the connection to the base plate is more difficult and the resistance against the impact of the milling cutter can be problematic for smaller diameters.

The minimum printable diameter in the SLM process is d=1mm, with cylinders having smaller diameters gaining insufficient connection to the building plate (200 μm–600 μm) or being damaged during powder recoating (800 μm). Employing the hybrid system, the smallest achievable cylinder diameter is d=1.4mm. Due to the low resistance of the specimens, thinner cylinders, reliably built by the SLM down 1.0 mm, break during the milling process.

Figure 12 depicts the deviation of the measured from the designed diameter for both the SLM and hybrid built parts. While for SLM built cylinders this deviation is in the range of 0.11 mm and 0.14 mm, it is significantly reduced to about 15 μm for the hybrid process, confirming that the hybrid manufacturing ensures better geometrical accuracy as provided by the sole SLM process.

#### 3.2.4. Overhanging Structures

As a consequence of insufficient heat dissipation, in SLM it is recommended to avoid overhanging structures without the use of support structures [53,54,55]. The hybrid manufacturing does not allow for the machining of horizontal structures underneath, in consequence the fabrication of classic constructed overhangs cannot be improved by the high speed milling process. For both reasons, the construction of overhanging structures should include self-supporting structures [29,56,57]. Apart from that, the length and the thickness of an overhanging structure are essential for the successfull manufacturing of unsupported overhangs.

Our study shows that overhanging structures can be built without support structures with a maximum lenght of l=5mm and a minimum thickness of d=0.3mm in the SLM process, concluding a design rule for unsupported overhangs as depicted in Figure A1. In addition, it can be specified that an overhang, supported by an inclined structure, ensures the heat dissipation and enables a surface finish with the hybrid manufacturing.

#### 3.2.5. Gap Width

The heat input in SLM is the decisive faktor for the gap width between two elements [58], while in the hybrid manufacturing approach it is given by the radius of the milling cutter adding the allowance for the finishing process for every flank of the gap.

With this, Equation (1) develops as a construction rule for the hybrid manufacturing of gap widths or notches. Due to the diameter of the used milling cutter of d=2 mm and the general finishing allowance of a=30μm, the minimum gap width for this study is b=2.06 mm using the hybrid manufacturing approach.

(1) $$b=d_{\mathrm{milling}\;\mathrm{cutter}}+2*a_{\mathrm{finish}}$$

**Figure 13:**
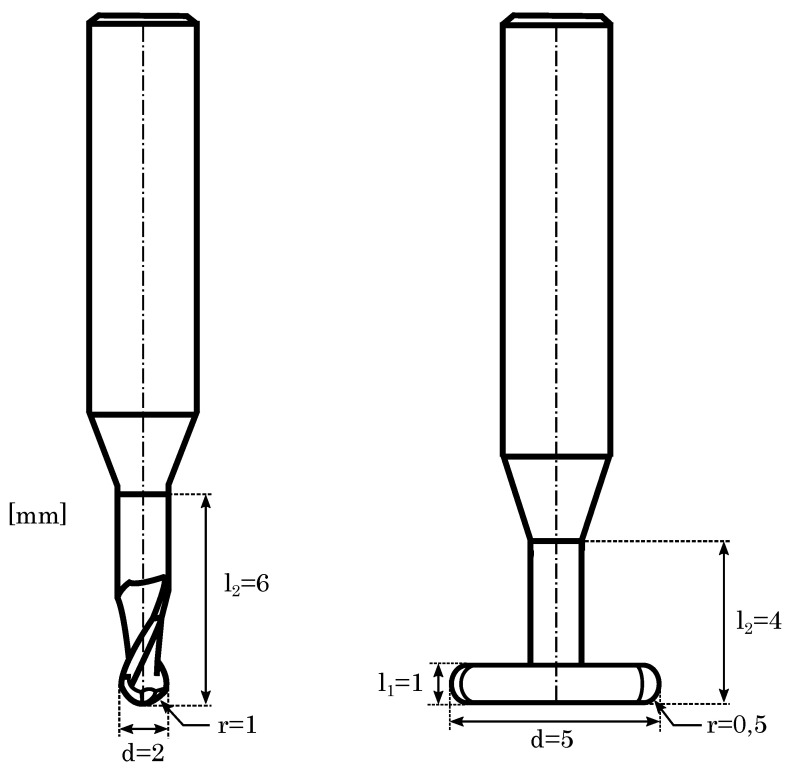
Dimensions of the 1 mm ball end mill and the T-slot cutter.

Within SLM, the minimum gap width can be defined by b=0.6 mm (maximum operated height h=3 mm). Due to heat dissipation, smaller widths get sealed or adhesion of powder particles deranges the aperture. Furthermore, it is founded that the rectangular transition at the bottom of the notch has not been machined completely, the milling cutter was not able to remove the entire allowance. By virtue of its dimensions, especially in view of the radius r (cf. Figure 13) and the three axis milling system, material transitions in build direction have to be adapted to this geometry. Thus, a continuative study, examining chamfers in build direction, has been carried out to define the limitations of the milling system for material transitions (cf. Section 3.2.10).

#### 3.2.6. Outer Diameter of Spheres

While spherical structures are, in general, contingent for SLM, challenges arise for manufacturing of complete spheres. The use of support structures is essential, because, compared to the manufacturing of inclined structures, the heat has to be dissipated across a very small base area with a quickly enlarging geometry [59,60].

For the hybrid manufacturing approach, the milling of sperical structures has to be divided in two sub processes. The overhanging structures are machined with the T-slot cutter, while the upwards facing surfaces get milled by the ball endmill to assure the best possible result in surface roughness.

**Figure 14:**
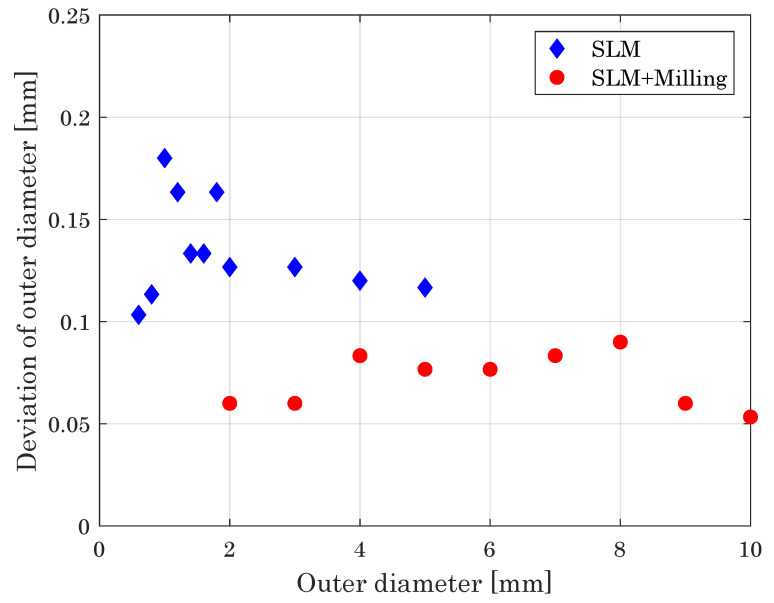
Deviation of outer diameter of spheres versus intended outer diameter, giving a measure of the dimensional accuracy of the processes.

As mentioned before, the heat dissipation determines the successfull manufacturing of outer spherical structures. Thus, the usage of support structures in the SLM process is elementary to avoid thermal deformation of the spheres in z-direction. Within the hybrid manufacturing, the remaining geometrical deformation is corrected by the milling cutter afterwards and provides a superior geometrical accuracy. As depicted in Figure 14, the deviation of SLM built parts lays between 0.1 mm and 0.18 mm, whereas the deviation of hybrid built parts can be diminished to underneath 0.1 mm in general.

For both processes, the minimum diameter of spheres with reliably built circular geometries can be realized down to d=2 mm, below this, the specimens get deformed by the thermal impact of the SLM process.

#### 3.2.7. Inner Diameter of Spheres

Analogue to the manufacturing of outer diameters, the use of support structures is necessary for the manufacuring of inner radii of spheres to guarantee a sufficient heat dissipation [61,62].

The hybrid manufacturing of inner diameters is, again, limited by the diameter of the milling cutter, as Equation (1) defines. Different to the manufacturing of notches, the spheres eliminate the necessity of an additional rounding in the lower part of the component, caused by the radius r of the milling cutter.

With that, the manufacturability of inner diameter of spheres is given at a minimum diameter of 1 mm in the SLM process and 2.06 mm, using the hybrid manufacturing approach and the 1 mm ball end mill (cf. Figure A1).

**Figure 15:**
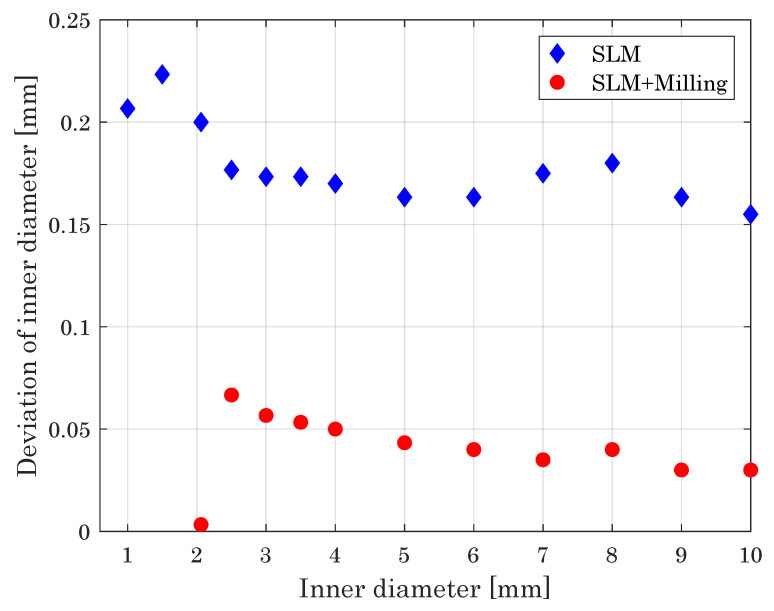
Deviation of inner diameter of spheres versus intended inner diameter, giving a measure of the dimensional accuracy of the processes.

**Figure 16:**
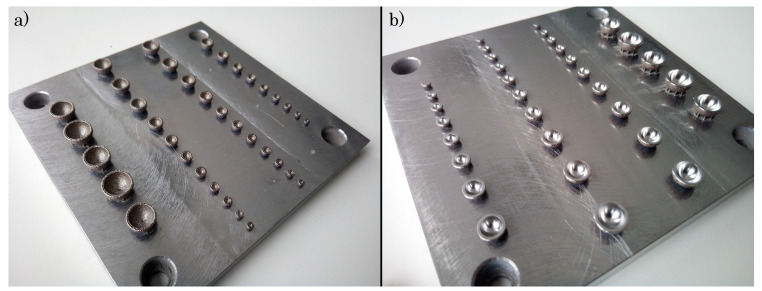
Comparison of (**a**) SLM-built and (**b**) hybrid built inner diameter of spheres.

As shown in Figure 15, the geometrical accuracy of the hybrid manufacturing approach exceeds the generated accuracy in the sole SLM. In addition, Figure 16 depicts the milled spheres in contrast to the SLM-built objects to distinguish the superior smoothness of the hybrid process. Furthermore, the results reveal that the minimum manufacturable inner diameter of 2.06 mm is fabricated reliably without high deviations.

#### 3.2.8. External Edges

For SLM, outer edges cannot be realized as sharp edges and it is generally recommended to use rounded edges for avoiding geometrical deviations [15]. In the hybrid approach, in addition, the impact of the milling cutter limits the fabrication of sharp edges. As seen before, the milling cutter can induce vibrations and may destroy the structure, turning around a sharp corner (cf. Section 3.2.2).

Nevertheless, as long as the minimum wall thickness is guaranteed (cf. Section 3.2.2), this hampering impact of the milling cutter is negligible and the fabrication of sharp edges can significantly be improved by the hybrid approach.

#### 3.2.9. Inner Angles

Material transitions, in general, should be rounded in the SLM process, conditioned by the laser additive process itself [63,64]. In the hybrid manufacturing the fabrication of inner angles is limited by the geometry of the milling cutter. Due to converging of the legs, inner angles lead to a unmillable geometry at a certain point, undercutting the diameter of the milling cutter.

**Figure 17:**
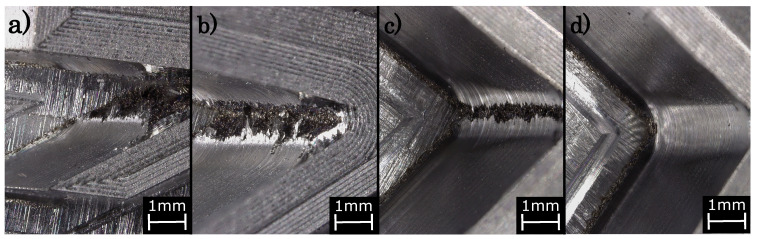
Microscope images of different inner angles with (**a**) 45${}^{\circ }$, (**b**) 60${}^{\circ }$, (**c**) 75${}^{\circ }$ and (**d**) 90${}^{\circ }$ after the milling process.

Figure 17 depicts different inner angles after the milling process. Apparently, the milling cutter is not capable to accurately machine angles below 75${}^{\circ }$. Only the 90${}^{\circ }$-angle can be completely milled, exhibiting an unconstructed rounding of the milling cutter.

As a consequence and as a design rule, inner angles have to be defined such that the vertex of the angle has to be rounded according to the diameter of the milling cutter (cf. Figure 13) as to guarantee a complete hybrid processing including an entire removal of the finishing allowance.

#### 3.2.10. Chamfers in Build Direction

Analogue to the manufacturing of inner angles, it is favourable to prevent sharp edges in build direction to minimize geometrical deviations [63,64]. By means of hybrid manufacturing, the milling cutter limits the creation of sharp chamfers by virtue of his geometry, as shown in Figure 13, illustrating the dimensions of the used ball end mill and the T-slot cutter. In the three-axis milling system, the radius r defines the minimal rounding of material transitions in build direction, especially for directly rectangular transitions.

While for sole SLM the manufacturing of chamfers in build direction is feasible, for the hybrid approach the radius of the milling cutter defines the minimum rounding of the material tranition. In the range of 90${}^{\circ }$–105${}^{\circ }$ the transition has to be rounded minimal in this radius, from 105${}^{\circ }$ the transition gets manufactured completely, including an entire removal of the finishing allowance.

#### 3.2.11. Bore Holes

The manufacturing of bore holes is, with particular respect to the integration of cooling channels, one of the most interesting application of the SLM [65,66,67]. Geometrically, bore holes incorporate overhanging parts and to circumvent the necessity of support structures at higher diameters, the use of self-supporting structures is recommended to avoid thermal deformation [14,68].

In hybrid manufacturing, the post-processing is, similar to the manufacturing of outer spheres, divided in two steps, milling the upper surfaces with the ball end mill, while the overhanging part gets machined by the T-slot cutter. Hence, the minimum diameter is defined to d=5.06 mm, following Equation (1) with a diameter of d=5 mm for the T-slot cutter. However, a non millable part of the bore hole remains at the top, depending in its pecularity on the geometry of the bore hole.

**Figure 18:**
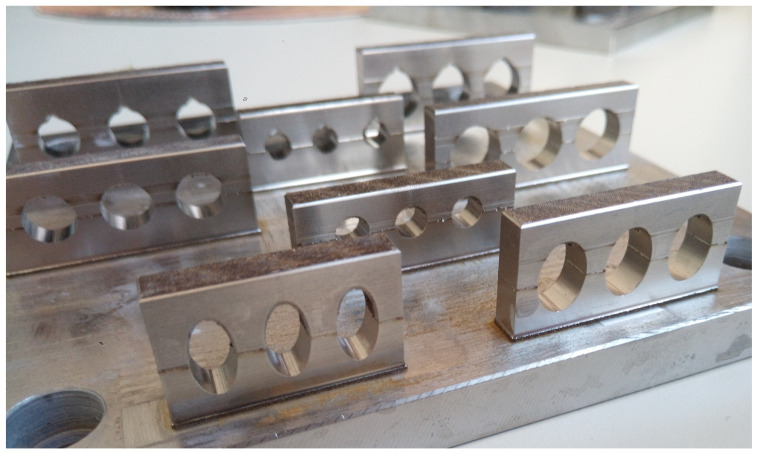
Different types of hybrid built bore holes.

For the study of the manufacturing of bore holes, different geometries are examined, embracing the classic circular hole, an elliptic hole and a self-supporting geometry, as depicted in Figure 18. In SLM, the classic circular bore hole has to be supported at a diameter larger than 7.5 mm, excluding the usage as a channel. The elliptic and the self-supporting geometry enable a manufacturing without the necessity of support structures independent on the diameter of the bore hole.

The hybrid manufacturing approach is, as mentioned before, limited to a minimum diameter of d=5.06 mm, remaining a non millable part at the top of the bore hole depending on the geometry. The self supporting geometry and the elliptical holes converge rapidly and the non millable part increases in comparison to the the circular bore hole.

#### 3.2.12. Inner Radius of Cylinders (Channels)

Analogue to the manufacturing of notches, the inner radius of cylinders depends, using the hybrid manufacturing approach, on the diameter of the used milling cutter, following Equation (1). Different to the manufacturing of notches, at the inner radius of cylinders, the expulsion of powder can lead to problems and increased signs of wear at the flank of the milling cutter.

In the SLM process, the heat input of the laser is decisive for the manufacturability of channels. They can get sealed by the thermal influence of the surrounding material and adhesion of powder particles can derange the aperture.

For confirming the specification in Equation (1), studying the performance of major cutters and observing the powder expulsion, the manufacturing of inner radii has been examined with the 1 mm ball end mill and the T-slot cutter. As depicted in Figure 13, the T-slot cutter has a diameter of d=5 mm in a completely plane geometry, increasing the difficulty of the powder expulsion.

In the SLM process, the minimum inner diameter is experimentally evaluated with d=0.6 mm, smaller channels get sealed or choked by powder adhesion.

In the hybrid process, the Equation (1) is confirmed with both milling cutters, so the minimum diameter is determined with d=2.06 mm for the ball end mill and with d=5.06 mm for the T-slot milling cutter. Furthermore, it is observed that the powder expulsion does not disturb the milling process and the tool wear is not increased significantly by the finishing work in this geometry.

### 3.3. Industrial Application—Injection Mould

For the exemplification of the potential of this hybrid approach, an injection mold for a charge socket for an electro mobile has been constructed, according the afore discussed design rules and manufactured. Using the advantages of the hybrid manufacturing, injection molds are one of the most interesting industrial application.

With the hybrid addtive manufacturing, injection molds can be built including cooling channels on the one hand and, with a good surface quality and a high geometrical accuracy on the other hand. Due to this, the quality of the die cast components is ensured and the heat dissipation is speeded up, lowering the process duration.

**Figure 19:**
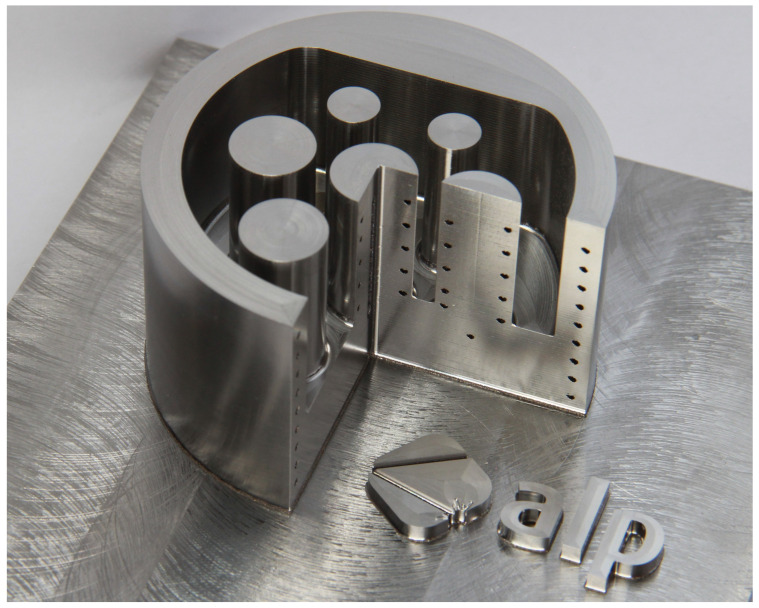
Injection mold of a charge socket for an electro mobile with a spared part, confirming the conformal cooling.

The injection mould is manufactured with a spared part for exhibiting the cooling channels, as shown in Figure 19. As visible, a conformal cooling with a diameter of d=2 mm for the cooling channels was realized, warranting an optimal heat dissipation in all parts of the injection mold. Further, the milling cutter provides a smooth surface, disabling asperities and with that, assuring an improved quality of the die cast components.

The manufacturing of such an unique component can only be permitted by this new manufacturing technique, combining the two fabrication processes. It synergises the geometrical freedom of the additive manufacturing with the surface quality and the geometrical accuracy of the milling process.

In regard to the quality of the die cast components, the surface roughness of an injection mould is one of the decisive factors for the process. Thus, injection molds get manufactured by conventional milling so far, ensuring a high surface quality. Though, the objective of this study is to identify design rules, it can be stated that, in general, the surface roughness Ra of the hybrid approach is better than 1μm for vertical and horizontal surfaces [34]. In case of curved geometries, like shown in Figure 19, this value raises slightly to Ra = 2μm.

## 4. Conclusions

We have evaluated fundamental properties of a hybrid SLM process, which encompasses selective laser melting as an additive process and high speed milling as a subtractive process. Though the milling process takes place within the powder bed, introducing additional splints of the milled steel and the cutter, used powder exhibits comparable properties in particle shape and size as the pristine powder after sieving, i.e., oversized particles with a length of below 0.5 mm can successfully be removed in the recycling chain of the powder. In addition, during the SLM process we observe no adverse impact on accessible geometrical shapes and parts of these oversized particles.

With respect to design engineering, based on the distinctive features of the hybrid additive manufacturing approach, design rules for basic elements like walls, cylinders, angels, inclinations, overhangs, notches, channels, inner and outer radii of spheres, chamfers in build direction, and holes of different structures have been derived and compared to the sole selective laser melting process. The milling process itself, with regard to its specific properties and limitations in design engineering points, originating from the 3-axis milling system, was investigated. Moreover, the geometrical deviations, contingent on the different shapes of the elements, have been determined and with that, the advantages of the hybrid manufacturing approach in comparison to the sole SLM process have been carved out.

Concluding, the specified design rules are consolidated in a comprehensive catalogue for the ultimate support of design engineers to purposefully apply hybrid additive manufacturing. With that, the hybrid approach can be exploited to its full capacity as well as the advantages of this particular system can be capitalised and the existing disadvantages of the sole SLM process can be eliminated. However, by defining the constructional limitations of reliably reproducable parts, common drawbacks of quality assurance issues concerning the acceptance of AM processes as industrial procuction technology are overcome.

Exemplifying the potential of this particular hybrid approach, an injection mold with conformal cooling for a charge socket of an electro mobile has been manufactured. The component summarizes the advantages of both parts of the hybrid manufacturing. Due to the freedom of design in the selective laser melting, an injection mold of every shape can be built, integrating a conformal cooling in a single fabrication step. By virtue of the high speed milling system, the surface finishing can be assured, providing a good quality of the die cast components.

## Figures and Tables

**Table 1:** Process parameters SLM.

	Laser Power [W]	Scan Speed [mm/s]	Hatch Distance [μm]
Area	320	700	0.12
Contour	320	1400	—
Support	320	700	0.12

**Table 2:** Milling process parameters.

	Z-Pitch [mm]	Spindle Speed [rot/s]	Feed Rate [mm/min]
Roughing cutter	0.15	30,000	2000
End mill	0.1	30,000	1600

**Table 3:** Chemical composition of Maraging-steel powder.

Element	Fe	Ni	Co	Mo	Ti	Cr	Mn	Si	Al	C	S
wt%	Balance	17–19	8.5–9	4.5–5.2	0.6–0.8	≤0.3	≤0.1	≤0.1	0.05–0.15	≤0.03	≤0.01

## Data Availability

Not applicable.
